# A 33-year diagnostic odyssey in an Ashkenazi Jewish patient with Aicardi-Goutières syndrome

**DOI:** 10.1016/j.jacig.2025.100400

**Published:** 2025-01-03

**Authors:** Oskar Schnappauf, Hongying Wang, Ivona Aksentijevich, Daniel L. Kastner, Ronald M. Laxer

**Affiliations:** a Department of Natural Sciences and Institute for Functional Gene Analytics, Bonn-Rhein-Sieg University of Applied Sciences, Rheinbach, Germany; b Inflammatory Disease Section, National Human Genome Research Institute, National Institutes of Health, Bethesda, Md; c The Hospital for Sick Children and St Michael’s Hospital, Division of Rheumatology, Departments of Paediatrics and Medicine, GRIID Program, University of Toronto, Toronto, Ontario, Canada

**Keywords:** Aicardi-Goutières syndrome, interferonopathies, *SAMHD1*, genetic testing, carrier screening, copy number variation

## Abstract

The critical need for awareness and genetic testing of the *SAMHD1* deletion in Ashkenazi Jewish patients is highlighted owing to its relatively high carrier frequency. Early detection can prevent severe disease complications through targeted therapy.

The detection of exogenous DNA is a key component of human immunity, primarily mediated by the cyclic guanosine monophosphate–AMP synthase (cGAS) and stimulator of interferon genes (STING) pathway. cGAS detects cytosolic double-stranded DNA, leading to production of cyclic guanosine monophosphate–AMP (cGAMP), which activates STING, initiating a signaling cascade that triggers a type I interferon response. Mutations in genes involved in this pathway, including SAM and HD domain containing deoxynucleoside triphosphate triphosphohydrolase 1 (*SAMHD1*), have been associated with disturbed self-DNA sensing and metabolism, resulting in constitutive activation of the cGAS-STING pathway.^1^ SAMHD1, with deoxyribonucleotide triphosphatase activity, regulates cellular deoxyribonucleotide triphosphate levels to inhibit early-stage viral replication, and supports genome integrity, responding to double-strand breaks and stalled replication forks.^2^ Defective *SAMHD1* results in elevated cellular deoxyribonucleotide triphosphate levels, unchecked spontaneous DNA damage, and release of self–double-stranded DNA, subsequently causing uncontrolled activation of the cGAS/STING signaling pathway.^3^

Aicardi-Goutières syndrome (AGS) is a monogenic but genetically heterogeneous (AGS1-9) systemic autoimmune disorder characterized by significant elevation of type I interferon levels in peripheral blood and cerebrospinal fluid, with early manifestations including progressive encephalopathy and chilblain-like skin lesions. The disorder may present at birth, resembling a congenital infection with neurologic impairments, or it may emerge in the initial months of life. After the first weeks of life, most affected infants present with severe encephalopathy characterized by extreme irritability, loss of skills, and decrease in head size. AGS5 is caused by biallelic loss-of-function variants in SAMHD1.^4^

We present the case of a 33-year-old man who was born to nonconsanguineous Ashkenazi Jewish parents and has 1 healthy brother (Fig 1, *A*). He was investigated for a long-standing illness that included neurologic impairments and systemic inflammation. He was initially diagnosed with cerebral palsy, and later in childhood, he developed severe chilblain lesions and inflammatory subcutaneous nodules (Fig 1, *B*). Brain magnetic resonance imaging revealed bilateral basal ganglia lacunar infarcts with periventricular white matter changes. Ependymal calcifications were identified in the lateral ventricles by using a computed tomography scan when the patient was 13.5 years old (Fig 1, *C*). Lesional skin biopsy samples showed evidence for panniculitis with lipoatrophy. Although alpha-1-antitrypsin deficiency can be associated with panniculitis, alpha-1-antitrypsin levels were not measured in this patient. Magnetic resonance imaging identified abnormal signals of subcutaneous fat in the patient’s upper and lower extremities, with multiple subcutaneous nodules, apparent thickening of the distal esophagus, and inflammatory infiltration of muscles. Muscle biopsy samples showed global atrophy of the muscle with fatty infiltration and some increased signal in several muscles, compatible with a diagnosis of myositis. Treatment with glucocorticoids and immunosuppressants did not lead to resolution of the patient’s inflammatory state. He developed progressive severe contractures and is wheelchair bound. The patient has limited language development. Collectively, his clinical findings indicated a disorder in the spectrum of primary interferonopathies, including AGS.

**Fig 1:**
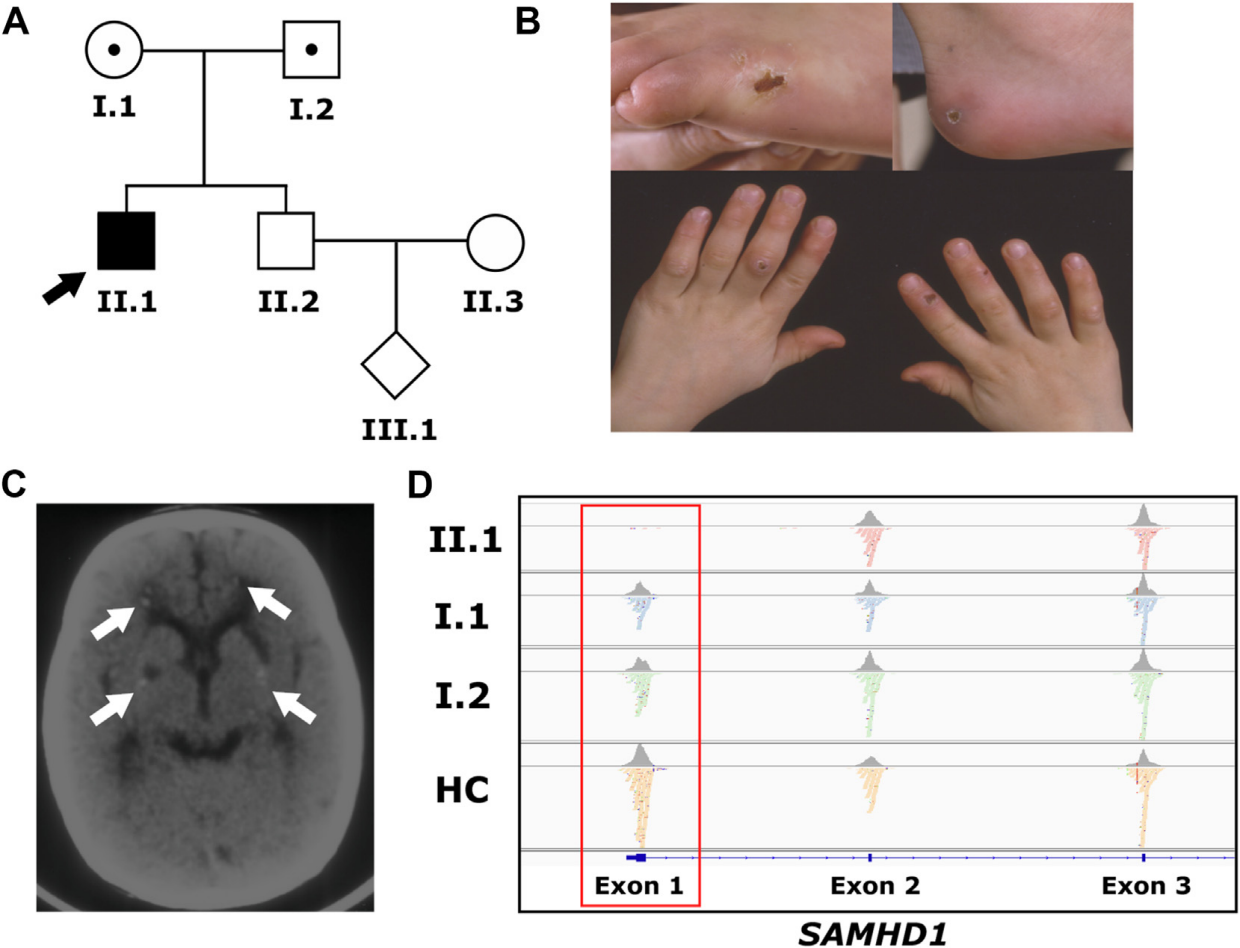
Family pedigree and patient presentation. **A,** Pedigree chart illustrating inheritance pattern of *SAMHD1* deletion. Carriers marked with central circle (carrier status of II.3 is unknown). Patient indicated with black arrow. **B,** Chilblain-like skin lesions observed on the patient’s feet and hands. **C,** Computed tomography scan showing ependymal calcifications in the lateral ventricles. **D,** Integrated Genome Viewer visualization of *SAMHD1* exons 1 to 3 in the patient (II.1), father (I.1), mother (I.2), and healthy control (HC).

Targeted next-generation sequencing was performed on the patient and his parents (see the Methods section in the Online Repository at www.jaci-global.org). No pathogenic single-nucleotide variants or indels were identified in the AGS-associated genes or other candidate genes. The copy number variation (CNV)-calling algorithm Atlas-CNV identified a homozygous deletion encompassing exon 1 of *SAMHD1* in the patient and heterozygous deletions in the parents (see Fig E1, *A* in the Online Repository at www.jaci-global.org).^5^ The deletion was visually confirmed using Integrated Genome Viewer (Fig 1, *D*). PCR with primer flanking the deleted region generated an approximately 500-bp product only in the patient and his carrier parents but not in the patient’s healthy brother or a healthy control sample (Fig 2, *A* and see Fig E1, *B*). Sanger sequencing of the amplification product identified a large (∼9-kb) deletion of genomic DNA (GRCh38: chr20: 36948791_36957774del) comprising exon 1 of *SAMHD1* (Fig 2, *B*). Quantitative RT-PCR analysis showed a significant reduction in *SAMHD1* expression in the patient and an approximately 50% reduction in the parents (Fig 2, *C* [*left panel*]). The patient’s brother showed *SAMHD1* expression levels comparable to those in a healthy control sample. Expression of the interferon-regulated gene *ISG15* showed increased expression in the patient compared with in his family members and healthy controls (Fig 2, *C* [*right panel*]). A custom-designed NanoString-RNA expression array of 31 interferon-regulated and other inflammatory genes showed upregulation of interferon-stimulated genes in the peripheral blood of the patient versus in the healthy controls (Fig 2, *D*).

**Fig 2:**
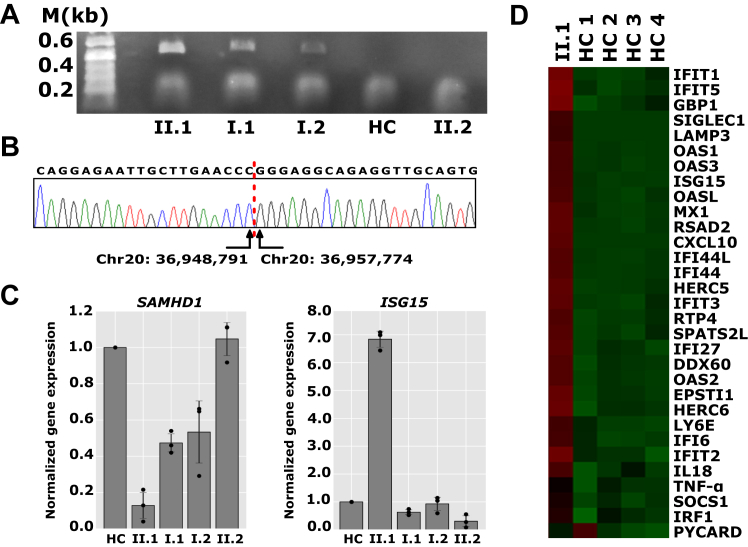
Genetic analysis and expression profiling of SAMHD1. **A,** PCR amplification of *SAMHD1* fragment from genomic DNA of family members and healthy control (HC). An approximately 500-bp PCR product is generated in the patient and carrier parents but not in the patient’s brother or HC. **B,** Sanger sequencing of PCR fragment shows breakpoints of deletion. **C,** Normalized expression of *SAMHD1* and *ISG15* in the family and HC. **D,** NanoString analysis of interferon-stimulated genes in the patient and HCs.

The 9-kb deletion includes the *SAMHD1* promoter and exon 1, eliminating the start codon and likely resulting in absent or disrupted protein production. This CNV, which has been reported in other patients with AGS, has a minor allele frequency of 1.35 × 10^−4^ in gnomAD SV v4.1.0, with the highest frequency (3.15 × 10^−3^) among individuals of Ashkenazi Jewish descent (see Table E1 in this article's Online Repository at www.jaci-global.org).^6^^,^^7^ This deletion is the most frequent pathogenic variant causing *SAMHD1*-associated AGS in the Ashkenazi Jewish population.^4^ The deletion is likely caused through nonallelic homologous recombination mediated by 2 highly homologous (83% sequence identity) Alu elements flanking the deleted region (see Fig E1, *C*).

At the time of the patient’s evaluation, his healthy brother was expecting a child with his Ashkenazi Jewish wife. Given the high carrier frequency of this pathogenic variant in the Ashkenazi Jewish population, determining the brother’s zygosity was crucial for proper risk assessment and counseling of the couple. The identification of heterozygous CNVs using read depth data from targeted next-generation sequencing (NGS) panels or exome data is hindered by biases inherent in the data (guanine-cytosine content, repetitive sequences, pseudogenes, and ambiguous mapping). We therefore performed PCR with flanking primers and expression analysis to exclude a carrier status in the patient’s brother (Fig 2, *A* and *C*).

In conclusion, our study underscores the critical importance for physicians and genetic testing providers to be aware of the described *SAMHD1* deletion. In patients suspected of having a primary interferonopathy, particularly in individuals of Ashkenazi ancestry, this deletion should be considered in NGS-based testing. Additionally, computed tomography imaging can serve as a complementary tool for the detection of calcifications, thereby aiding in the diagnosis. Because of the limitations of read depth–based CNV algorithms using NGS data for detecting individuals heterozygous for the deletion, alternative methods such as long-read sequencing, multiple ligation-dependent probe amplification, microarray, or quantitative RT-PCR, are recommended for carrier testing.

Early detection can enable tailored therapies (eg, JAK inhibitors, anifrolumab), potentially avoiding glucocorticoids and reducing disease complications.^8^ Given the relatively high frequency of this deletion in individuals of Ashkenazi Jewish descent, testing for the *SAMHD1* genomic deletion could be incorporated into carrier screening protocols for this population.^9^

## Disclosure statement

This work was funded by the 10.13039/100030692Intramural Research Program of the 10.13039/100000051National Human Genome Research Institute (grant HG200372-07).

Disclosure of potential conflict of interest: R. M. Laxer reports consulting with Sobi, Novartis, Eli Lilly Canada, Sanofi, and Akros Pharma, as well as royalties from UpToDate. The rest of the authors declare that they have no relevant conflicts of interest.
