# Supplementary material for: A 33-year diagnostic odyssey in an Ashkenazi Jewish patient with Aicardi-Goutières syndrome
Source: J Allergy Clin Immunol Glob. 2025 Jan 3;4(2):100400. doi: 10.1016/j.jacig.2025.100400 (PMC11791129; doi:10.1016/j.jacig.2025.100400)
Supplement: Supplementary data [file mmc1.docx]

**Supplementary Materials**

**Materials and Methods**

**1. Patient Information**
The patient was managed at The Hospital for Sick Children, Toronto, Canada. The patient was consented under the NHGRI Institutional Review Board approved protocol.

**2. Next-Generation Sequencing Panel**
Targeted next-generation sequencing (NGS) was conducted on 241 genes associated with autoinflammation and autoimmunity. The Twist Biosciences Target Enrichment Methodology and Library Preparation Technology were utilized to generate NGS libraries, which were sequenced on an Illumina MiSeq platform using 150 bp paired-end reads with version 3 chemistry. Sequence data were processed with CASAVA 1.8.2 and RTA 1.18.54. Alignment of sequence reads was performed using the Burrows-Wheeler Aligner (BWA) against the UCSC hg19 reference genome. The average read depth achieved was 96x. Variant calling, including single-nucleotide variants (SNVs) and small insertions/deletions (indels), was performed using the Genome Analysis Toolkit (GATK) with best practice parameters. The CNV-Atlas algorithm was employed to detect larger copy number variations (CNVs).

**3. PCR Amplification Using Flanking Primers**
Genomic DNA from the patient, parents, and a healthy control was amplified using the following primers: forward (5’-GCCAAGGCGTGTGAATCATC-3’) and reverse (5’-GAGAGCTCTTTGGCCCCATT-3’). Amplification was conducted using AmpliTaq Gold Fast PCR Master Mix (Thermo Fisher Scientific). Sanger sequencing was performed using the BigDye Terminator Cycle Sequencing Kit on a 3130xl Genetic Analyzer (Applied Biosystems), utilizing the same primers for sequencing. Data analysis was carried out with Sequencher software (Gene Codes).

**4. Quantitative RT-PCR**
RNA was extracted using the PAXgene Blood RNA System (Qiagen). A total of 250 ng of RNA was reverse-transcribed using SuperScript III Reverse Transcriptase (Thermo Fisher Scientific). Quantitative RT-PCR (qRT-PCR) was performed in duplicate using TaqMan Master Mix II with UNG (Thermo Fisher 4440038) on a ViiA 7 Real-Time PCR System. The following probes were used: GAPDH (Hs99999905_m1), ISG15 (Hs00192713_m1), and SAMHD1 (4331182 Hs00210019_m1). Expression levels were normalized to GAPDH, and data analysis was performed using the Applied Biosystems software.

**5. Nanostring analysis**

The expression analysis of 31 interferon-regulated genes was conducted using the nCounter Analysis System from NanoString Technologies. A total of 100 ng of RNA was combined with specific capture and reporter probes and subsequently hybridized on the nCounter Prep Station. The resulting data was analyzed utilizing both nSolver software and MATLAB.

**Supplementary figures**

Supplementary figure 1: Identification and characterization of a 9kb deletion in *SAMHD1*

A: CNV-Atlas read depths over genomic region chr20:35579839-35580046 (hg19) for 48 individuals, patient (P), his father (F) and mother (M).

B: Representation of SAMHD1, location of 9kb deletion, position of primer used for amplification, resulting in ~500bp product if deletion is present.

C: Alignment of AluSc (Query) and AluSc8 (Sbjct) using the NCBI BLAST algorithm. Intronic breakpoint at position chr20: 36,948,791 overlaps the AluSc element (chr20:36,948,587-36,948,894), while upstream breakpoint at chr20: 36,957,774 falls within the AluSc8 repeat (chr20:36,957,567-36,957,877).

**Supplementary tables:**

Supplementary Table 1: Comparative analysis of the patient (this report) with the patient described by Leshinsky-Silver et al. (2010).

| Characteristic | Patient this report | Patient reported in Leshinsky-Silver et al. |
| --- | --- | --- |
| Age at Diagnosis | 33 years | 13 months |
| Ethnicity | Ashkenazi Jewish | Ashkenazi Jewish |
| Genetic Mutation | Homozygous 9kb deletion encompassing exon 1 of *SAMHD1* | Homozygous 9kb deletion encompassing exon 1 of *SAMHD1* |
| Neurological Involvement | Cerebral palsy, severe contractures, limited language | Global developmental delay, choreoathetoid movements, poor eye contact |
| Systemic Involvement | Systemic inflammation, panniculitis, myositis | Liver dysfunction, anemia, failure to thrive |
| Mitochondrial DNA Deletions | Not reported | Multiple mitochondrial DNA deletions |
| Chilblains Present | Yes | Not mentioned |
| Inflammatory Markers | Increased ISG15 and interferon signature in blood | Not mentioned (CSF analysis not performed) |
| Brain Imaging Findings | Bilateral lacunar infarcts, white matter changes | Extensive white matter destruction, delayed myelination, cortical atrophy |
| Calcifications on Imaging | Ependymal calcifications in the lateral ventricles | Periventricular calcifications in fetuses, not in the baby |
| Outcome | Wheelchair-bound, severe contractures, progressive systemic inflammation | Died at 15 months due to respiratory insufficiency |
